# Chronic diseases in pregnant women: prevalence and birth outcomes based on the SNiP-study

**DOI:** 10.1186/1471-2393-14-75

**Published:** 2014-02-19

**Authors:** Ines Kersten, Anja Erika Lange, Johannes Peter Haas, Christoph Fusch, Holger Lode, Wolfgang Hoffmann, Jochen Rene Thyrian

**Affiliations:** 1 Institute for Community Medicine, Section Epidemiology of Health Care and Community Health, Ellernholzstr. 1-3, 17475 Greifswald, Germany; 2 Department Neonatology and Pediatric Intensive Care, Hospital for Pediatrics, University of Greifswald, Ferdinand-Sauerbruchstr, 17489 Greifswald, Germany; 3 German Center for Neurodegenerative Diseases (DZNE), Greifswald, Germany; 4 Department of Pediatrics, Mc Master University, Division of Neonatology, West Hamilton, Ontario, Canada; 5 German Center for Rheumatology in Children and Adolescents, Garmisch-Partenkirchen, Germany

**Keywords:** Chronic disease, Pregnancy, SNiP-study

## Abstract

**Background:**

The subject of “pregnancy and disease” is of particular importance for maternal well-being and neonatal outcomes. The international literature has focused on acute diseases during pregnancy; however, there are only a few studies investigating chronic diseases in pregnant women. The focus of this study is on diseases of women in childbearing age that are not related to the pregnancy. The objective of the paper is to deliver population based prevalences of chronic dieases in childbearing women and compare the two groups of chronically ill women and healthy women in detail regarding sociodemography, peri- and prenatal parameters and birth outcomes.

**Methods:**

Data of n = 5320 childbearing women were evaluated in the context of the population-based Survey of Neonates in Pomerania (SNiP). Data were obtained via face-to-face interviews, self-applied questionnaires, and abstraction from medical records at the time of giving birth. Sociodemographic and health status data were assessed, including chronic diseases that were taken out of medical records. A comprehensive set of pre- and perinatal varaiables were assessed.

**Results:**

In the SNiP, every fifth pregnant woman suffers from at least one chronic disease, and higher prevalence rates have been reported in the literature. There was a significant difference between chronically ill women and healthy women in age, education and income. Prenatal complications were more frequent in the healthy group than in the chronic disease group. Women with chronic diseases delivered by Cesarean section more frequently than women in the healthy group. Every tenth woman with at least one chronic disease gave birth to a premature infant, while only one in every 13 woman in the healthy control group gave birth to a premature infant.

**Conclusions:**

This analysis is the first population-based study in which all chronic diseases could be taken into consideration. The population-based prevalences rates in the SNiP data are consistently lower than those found in the literature. There are differences between chronically ill women and healthy women in peri- and prenatal variables as well as birth outcome on the population level. However, they are less frequent than expected and further analyses are need focusing on specific diseases.

## Background

Chronic diseases can influence the course of pregnancy and may have lasting effects that manifest at and after birth. Therefore, it is not surprising that women with chronic diseases are often anxious about pregnancy. Fortunately, due to medical progress and detailed pregnancy planning in collaboration with specialists, it is rarely necessary to advise against pregnancy. For instance, 100 years ago, women with multiple sclerosis were advised against pregnancy; however, in subsequent decades, studies demonstrated that this disease may enter into temporary remission during pregnancy, and it is no longer considered a contraindication [1]. Another example is diabetes mellitus; prior to the introduction of insulin in 1922, patients with diabetes mellitus were considered to have a worsened pregnancy prognosis [2]. Epilepsy is also no longer a contraindication according to the “Deutsche Arzneimittelkommission” published in 1984. Today, pregnancy is contraindicated for some women with congenital heart failure or pulmonary hypertension (such as, Eisenmenger’s syndrome, primary pulmonary hypertension, secondary vascular pulmonary hypertension) [3,4].

Fertility may also be associated with chronic disease. Some (untreated) chronic diseases are known to cause a reduction in fertility, e.g., hypo- and hyperthyroidism or celiac disease. In these cases, other possibilities, such as intracytoplasmic sperm injection (ICSI) or in vitro fertilization (IVF), should be considered. In addition, women with epilepsy, polycystic ovarian (PCO) syndrome, rheumatic episode, endometriosis or vitamin B_12_ deficiency have reduced fertility [5]. A high risk pregnancy in a woman with a known disease requires medical care in specialized perinatal centers [6]. Severe maternal diseases of the cardiovascular and pulmonary systems (severe pulmonary hypertension, heart failure, aortic and mitral valve defects or widening of the ascending aorta in connection with Marfan syndrome), insulin obligatory diabetes, addictive disorders (alcohol, drugs) as well as chronic intrauterine infections (with CMV, HSV, HIV or toxoplasmosis) are indicative of a high risk pregnancy [6].

The subject of “pregnancy and disease” is of particular importance for maternal well-being and neonatal outcomes. The international literature has focused on acute diseases during pregnancy; however, there are only a few studies investigating chronic diseases in pregnant women. Studies have reported very different prevalence rates for chronic diseases among women in childbearing age. Hence, the focus of this study is on diseases of women in childbearing age that are not related to the pregnancy but may have an impact on pregnancy and birth outcomes. The following questions are examined in this study:

1. Is there a relationship between chronic disease and conception on the population level?

2. Are pregnancies more carefully planned by women with chronic disease compared to women without chronic disease?

3. Is the course of pregnancy more complicated in women with chronic diseases than for healthy women on a population-based level?

4. What is the relationship between chronic disease and birth outcome?

## Methods

### Data/study design

**Figure 1:**
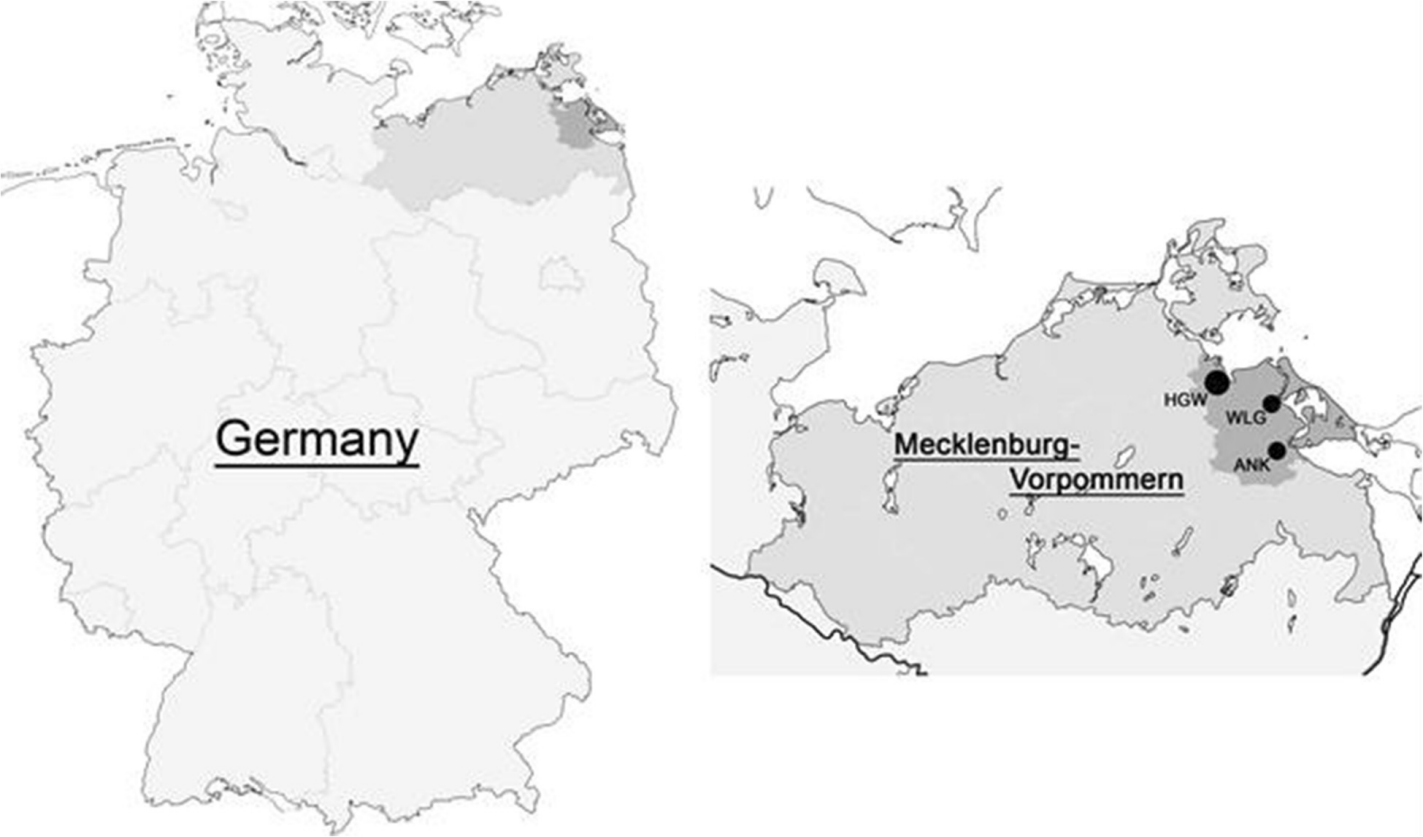
**Study region of “Ostvorpommern” (dark grey) with location of major Pediatric Hospitals (HGW: Hansestadt Greifswald, WLG: Wolgast, ANK: Anklam), based on **[7]**.**

Data were evaluated in the context of the population-based Survey of Neonates in Pomerania (SNiP)-The study was conducted in a rural county in Western Pomerania (“Ostvorpommern”) in North-Eastern Germany from 01.05.2002 to 30.11.2008 (see Figure 1). A detailed description of the study design is provided by Ebner et al. [7]. The study design was reviewed and approved by the Ethics Committee of the Board of Physicians, Mecklenburg-Western Pomerania at the University of Greifswald.

### Sample

**Figure 2:**
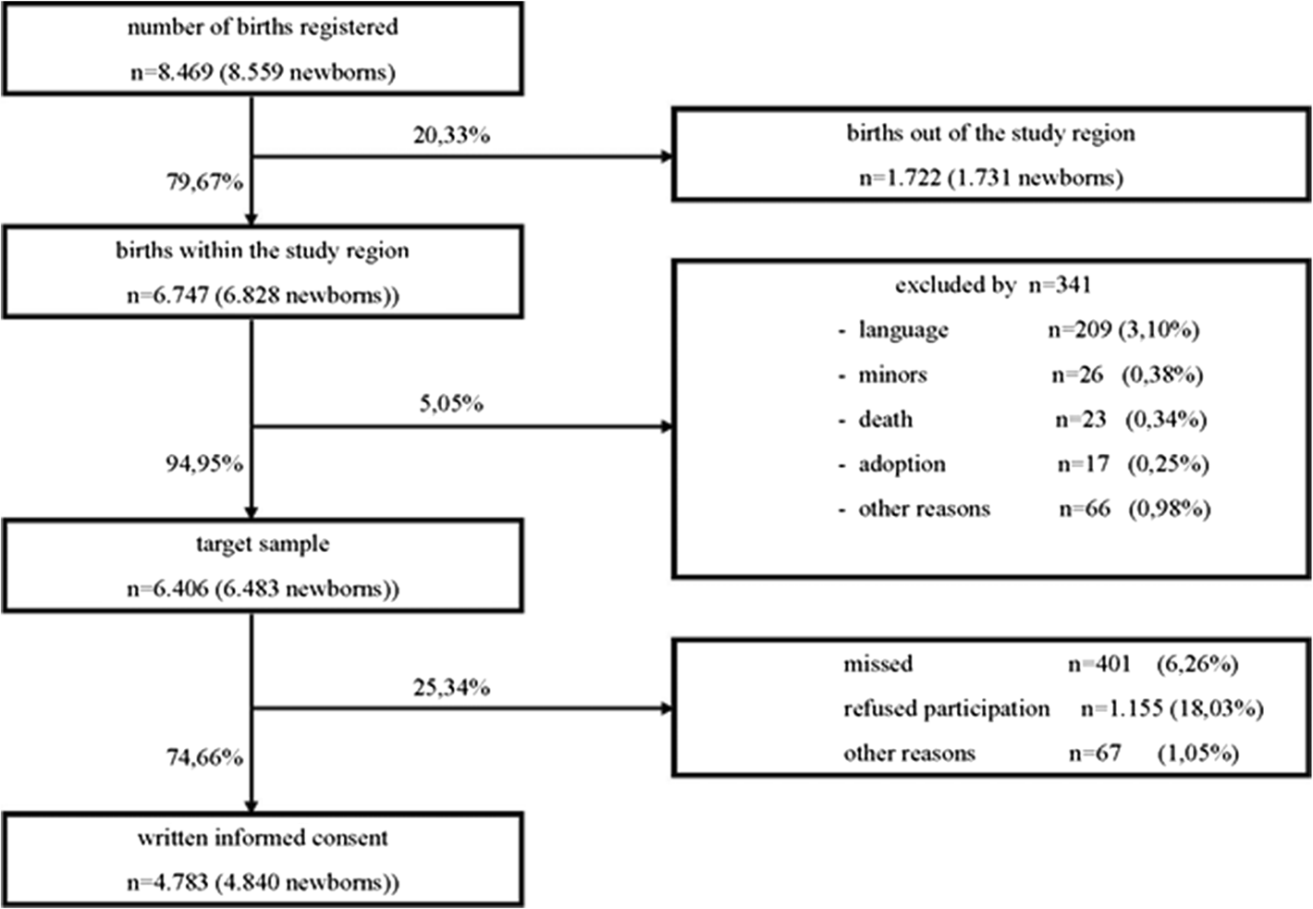
**Data and reasons of loss of potential participants in Survey of Neonates in Pommerania (SNiP), based on **[7]**, n: number of births.**

The SNiP population includes almost all pregnant women living in “Ostvorpommern” who gave birth between May 1st, 2002 and November 30th, 2008 since data were assessed on all maternity wards in this rural region and the percentage of giving birth in a stationary setting is above 98%. All pregnant women who fulfilled the inclusion criteria were asked for their written informed consent. Written informed consent was obtained from 75% of all women eligible for study participation. Figure 2 provides an overview of the number of births in the region, inclusion, exclusion criteria and reasons for non-participation. A non-responder analysis did not indicate relevant selection bias, so the sample under examination can be considered as population-based and representative.

### Data manipulation

Data were obtained via face-to-face interviews, self-applied questionnaires, and abstraction from medical records and stored in one data set per birth. Depending on the focus of each analysis, different numbers of data sets had to be analyzed. For questions referring to the newborn (perinatal parameters), every birth had to be analyzed (two data sets in twin births and three in triplet births). If the mother was the focus of the analysis, only one data set was analyzed in multiple births, for instance, in relation to diseases and prenatal parameters. In women who had more than one birth during the study period, all births were analyzed because sociodemographic changes and changes in maternal disease status could occur between births. To compare prenatal and perinatal parameters, it is useful to categorize women into groups according to number of live births and previous pregnancies. In category A, all women were primigravidas and primiparous. In category B, all participants were multigravida and primiparous, that is, women who had at least one abruption, extra-uterine pregnancy, abortion or stillbirth and who participated in SNiP with their first child. Women in category C were multigravida and multiparous with equal numbers of pregnancies and live births. In contrast, women in Category D were multigravida and multiparous with more pregnancies than live births. A fertility index was calculated for each category. The index is the difference between the gravidity and parity. A fertility index of 0 is optimal, an index of 1 is tolerable, and indices greater than 2 are pathological.

Furthermore, the period of maternal hospitalization due to complications during pregnancy, details of infections and acute diseases as well as vaginal hemorrhages during pregnancy and the birth modus (spontaneously, operative vaginal, operative abdominal, caesarean section (primary, secondary, emergently)) were assessed. The following perinatal parameters were also assessed: 5 minute Apgar score, first pH of the umbilical cord artery at birth, base Excess, extended primary care of the newborn, transfer of the newborn to a specialized center, gestational age, birth weight, birth length and head circumference.

### Statistical analysis

The software program SPSS for Windows (PASW, version 18.0) was used for the statistical analysis. Both descriptive and analytic statistical methods were used. Frequencies and proportions and the means and standard deviations were calculated where appropriate. Inference statistics were calculated using chi square tests according to Pearson’s coefficient and t-tests.

### Ethical approval

This study was approved by the Regional Committee for Medical Research Ethics at the University of Greifswald. All participants provided written informed consent.

## Results

### Prevalences of chronic diseases

**Figure 3:**
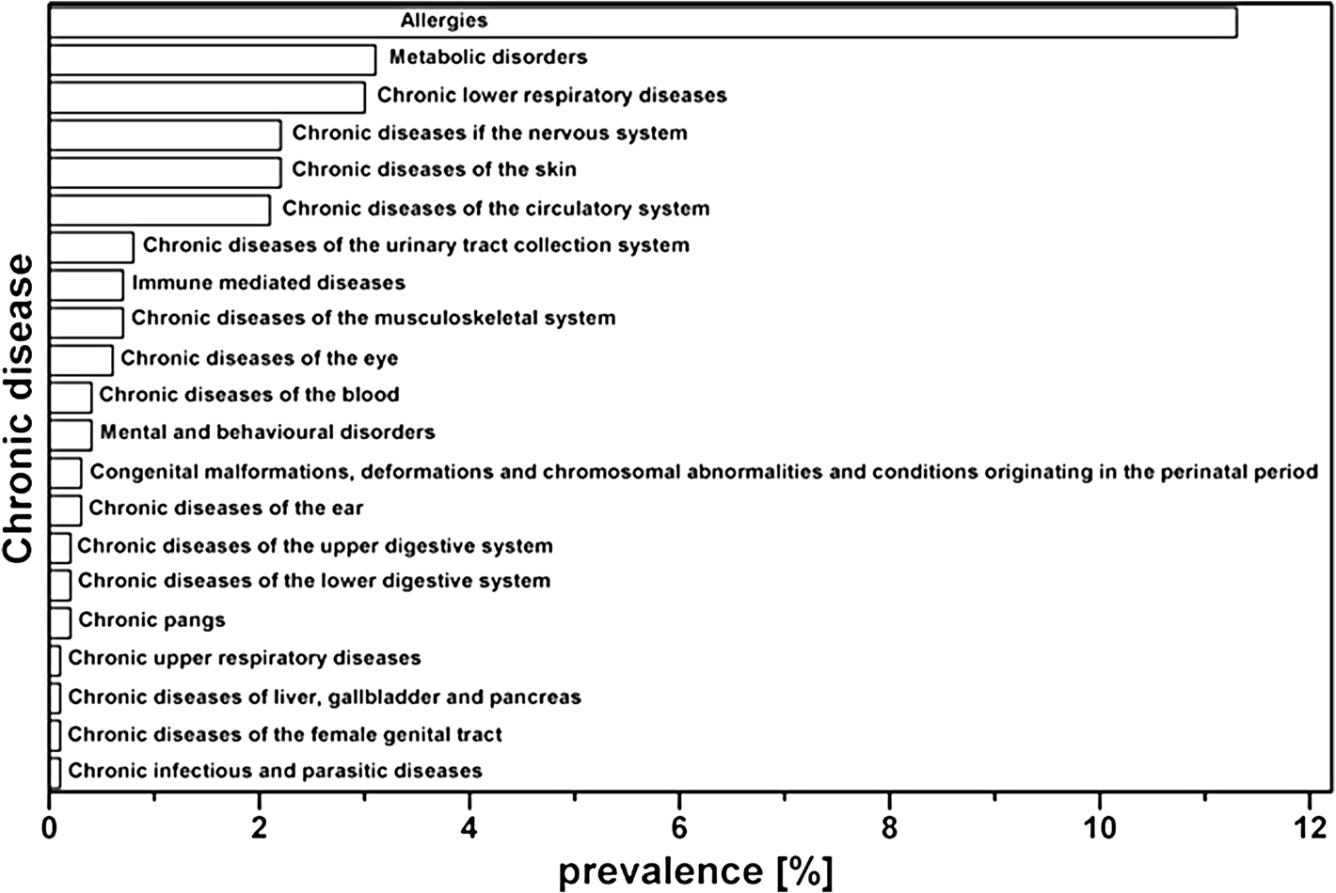
Prevalences of major chronic diseases in SNiP.

In n_1_ = 1141 SNiP participants, at least one chronic disease was reported (in the following descriptions, n_1_ are all women with chronic diseases and n_2_ are women without chronic diseases). More precisely, 738 women reported only one chronic disease, 285 reported two chronic diseases and 118 reported at least three chronic diseases. Table 1 shows the variety of chronic diseases reported in the study population. The specific diseases are listed with their ICD-10-codes; the absolute frequency (Hx (D)) and relative frequency are provided. The prevalences of chronic diseases among the SNiP participants are described by hx_1_ (D) and are summarized in Figure 3 for the main diseases. Because each chronically ill participant reported at least one disease, the total number of listed diseases is greater than 100%. The most frequently occurring diseases were allergies (prevalence of 11.3%), bronchial asthma (2.7%) and diseases of the thyroid gland (2.3%). Furthermore, diseases of the skin (2.2%), arterial hypertension (1.1%) and migraine (1.5%) showed relevant prevalences. In total, 11 women reported diabetes mellitus, 30 women reported epilepsy and 40 women reported at least one immune-mediated disease.

**Table 1:** **Chronic diseases in the SNiP with absolute frequency (Hx (D)) and relative frequency (hx**_**1**_**(D): applied to all n = 5330 women participated in SNiP, hx**_**2**_**(D): applied to n**_**1**_ **= 1141 women with chronic diseases)**

**Chronic disease**	**ICD-10-codes**	**Hx (D)**	**hx**_**1**_**(D) [%]**	**hx**_**2**_**(D) [%]**
**Chronic infectious and parasitic diseases**	**∑**	6	0.1	0.5
Tuberculosis	A15.- until A19.- and B90.9	3	0.1	0.3
Hepatitis	B18.- and K73.-	3	0.1	0.3
**Chronic diseases of the blood**	**∑**	21	0.4	1.8
Anemia	D50.- until D64.-	7	0.1	0.6
Other chronic diseases of the blood	D65.- until D69.- and D73.0	14	0.3	1.2
**Metabolic disorders**	**∑**	167	3.1	14.6
Hypothyreoidism	E03.- and E89.0	76	1.4	6.7
Hyperthyreoidism	E05.-	19	0.4	1.7
Other metabolic disorders	E00.- until E02.- and E04.- and E06.2 until E07.- and E89.1 until E89.9	27	0.5	2.4
Diabetes mellitus	E10.- until E14.- and O24.0 until O24.3	11	0.2	1
Other endocrine diseases	E20.- until E29.- and E31.- until E35.-	6	0.1	0.5
Alimentary deficiencies	E40.- until E46.- and E50.- until E64.-	4	0.1	0.4
Supernutrition	E65.- until E68.-	9	0.2	0.8
Other metabolic disorders	E70.- until E85.- and E88.- and E90.-	15	0.3	1.3
**Mental and behavioural disorders**	F00.- until F99.-	20	0.4	1.8
**Chronic diseases of the nervous system**	**∑**	116	2.2	10,.2
Multiple sclerosis	G35.-	1	0	0.1
Epilepsy	G40.-	30	0.6	2.6
Migraine	G43.-	80	1.5	7
Diseases of the cerebral nerves	G50.- until G53.-	1	0	0.1
Diseases of the PNS	G54.- until G64.-	2	0	0.2
Myopathies and paresis	G70.- until G73.- and G80.- until G83.- and R25.2	2	0	0.2
**Chronic diseases of the eye**	**∑**	33	0.6	2.9
Chronic disease of the iris and the ciliary body	H20.1 until H22.-	2	0	0.2
Chronic disease of the retina	H33.- until H36.-	4	0.1	0.4
Glaucoma	H40.- until H42.-	7	0.1	0.6
Disorders of optic nerve and visual pathways	H46.- until H48.-	2	0	0.2
Disorders of accommodation and refraction and other visual disturbances	H49.- until H54.-	18	0.3	1.6
**Chronic diseases of the ear**	**∑**	18	0.3	1.6
Chronic diseases of middle ear and mastoid	H65.2 until H65.9 and H66.1 until H69.x and H73.1 until H73.9	5	0.1	0.4
Chronic diseases of inner ear	H80.- until H83.-	1	0	0.1
Other disorders of ear	H90.- until H95.-	12	0.2	1.1
**Chronic diseases of the circulatory system**	**∑**	113	2.1	9.9
Hypotension	I95.- and R55.-	15	0.3	1.3
Hypertension	I10.- until I15.-	58	1.1	5.1
Chronic heart diseases	I31.- until I32.- and I34.- until I39.- and I42.- until I43.- and I50.- until I52.-	4	0.1	0.4
Cardiac conduction disorders	I44.- until I49.- and R00.-	16	0.3	1.4
Cerebrovascular diseases	I60.- until I69.-	2	0	0.2
Other vascular diseases	I26.- and I70.- until I73.- and I77.- until I80.- and I83.- until I89.- and I99.-	18	0.3	1.6
**Chronic upper respiratory diseases**	J31.- until J35.- and J37.- until J38.-	7	0.1	0.6
**Chronic lower respiratory diseases**	**∑**	159	3	13.9
COPD	J44.-	1	0	0.1
Asthma bronchiale	J45.-	144	2.7	12.6
Other chronic lower respiratory diseases	J40.- until J43.- and J47.- and J95.3 until J95.9 and J96.1 until J96.9	14	0.3	1.2
**Chronic diseases of the upper digestive system**	**∑**	9	0.2	0.8
GERD	K21.-	5	0.1	0.4
Other chronic diseases of the upper digestive system	K22.- and K29.3 until K29.7 and K31.1 until K31.5 and R12.- and Z90.3	4	0.1	0.4
**Chronic diseases of the lower digestive system**	**∑**	12	0.2	1.1
Chronic inflammatory bowel diseases	K50.- until K52.-	3	0.1	0.3
Other chronic diseases of the lower digestive system	K55.1 and K58.- and K59.- until K63.- and K92.8 until K92.9 and R15.-	3	0.1	0.3
Disorders of malabsorption	K90.- until K91.-	6	0.1	0.5
**Chronic diseases of liver, gallbladder and pancreas**	K70.-, K74.-, K76.-, K80.-, K81.1 until K83.-, K86.-	3	0.1	0.3
**Chronic diseases of the skin**	**∑**	118	2.2	10.3
Dermatitis	L20.-, L21.-, L24.-, L25.-, L26.-, L27.2 until L27.9, L30.-	105	2	9.2
Other chronic diseases of the skin	L55.- until L59.-, L70.- until L75.-, L80.-	13	0.2	1.1
**Immune mediated diseases**	**∑**	37	0.7	3.2
Psoriasis	L40.-	20	0.4	1.8
Urticaria	L50.-	4	0.1	0.4
Lupus erythematodes	L93.-, M32.-	1	0	0.1
Arthropathies	M05.- until M19.-, M79.0-	7	0.1	0.6
Diseases of the connective tissue	M30.-, M31.-, M33.- until M36.-, R60.-	4	0.1	0.4
Other immune mediated diseases	K75.4	1	0	0.1
**Chronic diseases of the musculoskeletal system**	**∑**	39	0.7	3.4
Dorsopathies	M40.- until M54.-	36	0.7	3.2
Chondropathies	M91.- until M94.-	1	0	0.1
Other chronic diseases of the musculoskeletal system	M95.- until M99.-	2	0	0.2
**Chronic diseases of the urinary tract collection system**	**∑**	43	0.8	3.8
Glomerular diseases	N01.- until N05.-	5	0.1	0.4
Renal tubulo-interstitial diseases	N11.- until N16.-	24	0.5	2.1
Urolithiasis	N20.- until N23.-	7	0.1	0.6
Other chronic diseases of the urinary tract	N18.-, N26.-, N28.-, N29.-, N30.1 until N33.-, N39.-	7	0.1	0.6
**Chronic diseases of the female genital tract**	**∑**	7	0.1	0.6
Noninflammatory disorders	N80.- until N90.-	5	0.1	0.4
Chronic inflammatory disorders	N70.1; N71.1; N72.-; N73.1; N73.4; N76.1; N76.3	1	0	0.1
Other chronic diseases of the femal genital tract	N91.- until N94.-	1	0	0.1
**Congenital malformations, deformations and chromosomal**	Q00.- until Q99.-, P00.-, P96.-	18	0.3	1.6
**abnormalities and conditions of the fetus originating in the perinatal period**			0	
**Chronic pangs**	G09.-, G44.-, R10.1 until R10.4, R51.-, R52.1 until R52.9	11	0.2	1
**Allergies**	**∑**	601	11.3	52.7
Systemic	J30.- and T63.4 and T78.1 and T78.4 and T88.7 and Z88.-	474	8.9	41.5
Dermatic	L23.- and L56.4	127	2.4	11.1

### Sociodemographic parameters

In our population-based study, n_1_ = 1141 women (with 1161 questionnaires) were identified as chronically ill (point prevalence 21.4%). In the chronic disease group, there were 20 twin births, and in the healthy group, there were 51 twin births and one triplet birth. N = 514 women had previously participated in SNiP with their baby’s siblings.

The distribution of sociodemographic parameters in the SNiP data is shown in Table 2. The average maternal age at the time of birth was 28 years for women with chronic diseases and 27 for healthy mothers; this difference is statistically significant (|t| = 4.294; p < 0.01). The age range was 14 to 45 years in the chronic illness group and 12 to 47 in the healthy group. In both groups, the majority of women were born in Germany. Most women were unmarried, followed by married cohabitating couples. Approximately half of the women in each group had received their secondary school certificate. In total, 36% of all women with chronic disease had acquired a higher level of education, whereas this proportion was only 30.2% in the control group (χ^2^ = 22,434; df =6; p < 0.01). In both the healthy control group and the chronic disease group, most participants had completed a professional training. A total of 40% of chronically ill women had a higher level of education compared to 34.9% of the healthy women (χ^2^ = 20.157; df =7; p < 0.01). The average monthly income was reported between 1,250 euros and 1,749 for both groups, but women with chronic diseases were more frequent in higher income categories than women without chronic diseases (χ^2^ = 20.157; df = 11; p < 0.05). Initially, we hypothesized that women with chronic diseases would plan their pregnancies more carefully than healthy women; however, the difference in this parameter was not statistically significant (χ^2^ = 5.652; df =2; p > 0.05).

**Table 2:** The distribution of sociodemographic parameters in participants of SNiP with significance verification (*: p < 0.05; **: p < 0.01; ***: p < 0.001)

**Variable**	**Characteristic**	**n**_**1**_ **= 1141**	**%**	**n**_**2**_ **= 4189**	**%+**
Age***	Mean	28.1	/	27.3	/
**[Years]**	Standard deviation	5.31	/	5.48	/
Age groups	< 20	52	4.6	258	6.2
	20-34	940	82.4	3465	82.7
	≥ 35	147	12.9	462	11
**Family status**	Married, cohabitating	403	35.3	816	19.5
	Married, separated	11	1	34	0.8
	Unmarried	573	50.2	1397	33.3
	Divorced	33	2.9	84	2
	Widowed	1	0.1	3	0.1
**Native country**	Yes	1114	97.6	4081	97.4
**Germany**	No	26	2.3	102	2.4
Graduation**	No graduation available	19	1.9	65	1.7
	Certificate of Secondary Education	100	9.7	521	14
	General Certificate of Secondary Education	518	50.4	1932	51.9
	Advanced technical college entrance qualification	46	4.5	165	4.4
	General qualification for university entrance	323	31.5	960	25.8
	Other graduation	19	1.9	65	1.7
	Presently still school-aged	2	0.2	16	0.4
Qualifications**	Presently articled	66	6.6	188	8.3
	No qualification available	56	5.6	191	8.4
	Completed traineeship	475	47.7	1093	48.3
	Completed business school	100	10	243	10.7
	Completed technical college	82	8.2	134	5.9
	Or university of cooperative education		0		0
	Completed college of higher education	66	6.6	123	5.4
	Completed university	143	14.4	279	12.3
	Other qualification	8	0.8	13	0.6
**Netto monthly**	Mode (1250–1749)	5	/	5	/
Income* [Euro]	< 500	87	9.6	232	11.6
	500-749	75	8.3	237	11.9
	750-999	82	9.1	199	10
	1000–1249	93	10.3	225	11.3
	1250–1749	137	15.2	278	13.9
	1750–1999	79	8.7	176	8.8
	2000–2249	67	7.4	135	6.8
	2250–2499	68	7.5	146	7.3
	2500–2999	95	10.5	173	8.7
	3000–3999	88	9.7	139	7
	4000–4999	25	2.8	36	1.8
	≥ 5000	7	0.8	21	1.1
**Pregnancy**	Yes, pregnancy was planned	707	62	1570	37.5
**Planned**	No, but no conception	199	17.4	517	12.3
	No, averted	117	10.3	219	5.2

Footnote: + Percentages do not always add up to 100% due to missing data.

### Prenatal parameters

There were no significant difference in fertility between women with and without chronic diseases, as determined by a calculation of the fertility index (χ^2^ = 3.141; df =3; p > 0.05).

**Figure 4:**
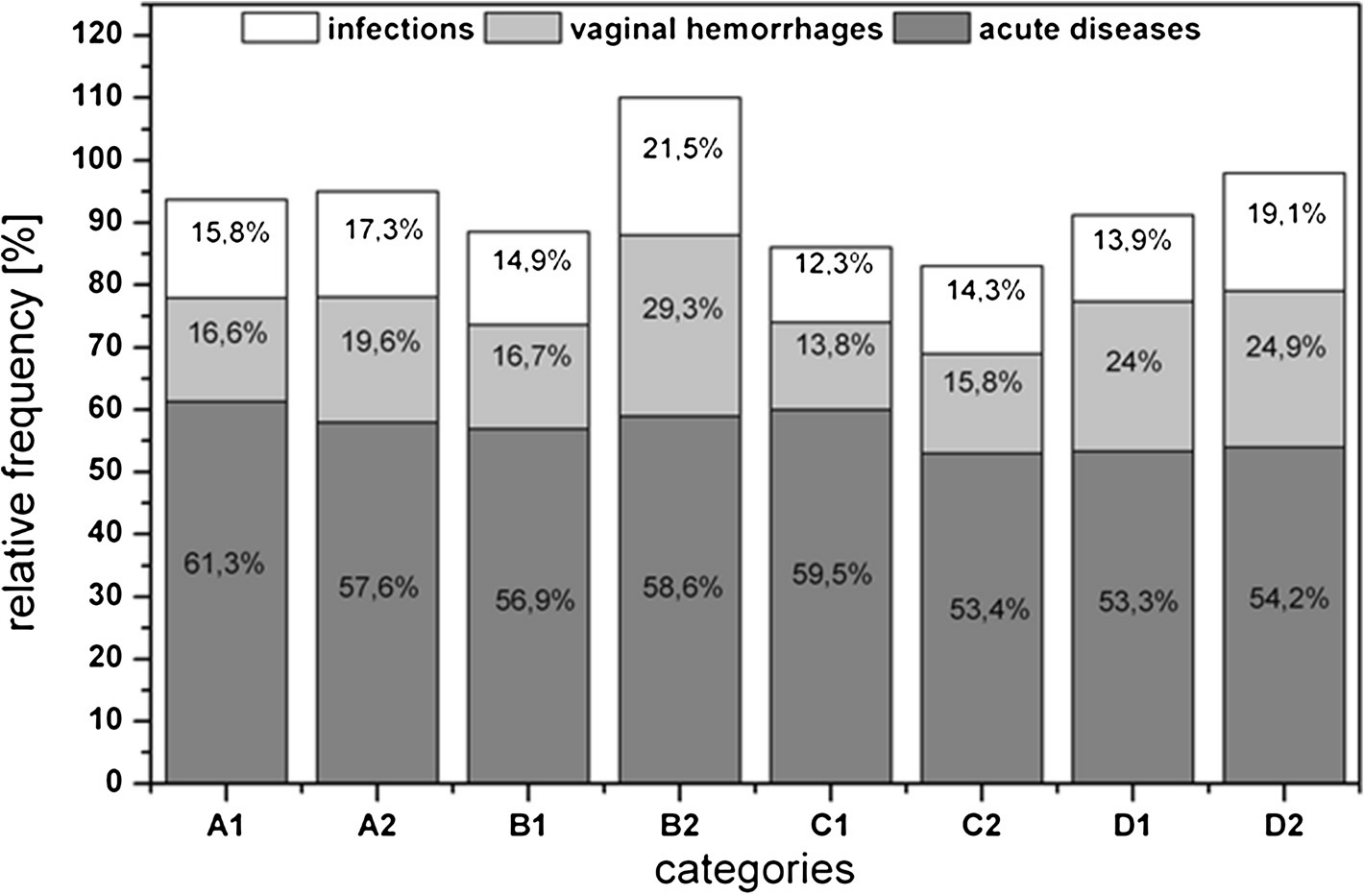
**Complications during pregnancy course plot against chronic diseases as well as pariety and gravidity. **A: primigravidae, primiparae, B: multigravidae, primiparae (women who had have at least one abruption, extrauterine pregnancy, abortion or stillbirth and who participated in SNiP with their first child), C: multigravidae, multiparae (the number of pregnancies correlates with the number of life births), D: multigravidae, multiparae (women who had have at least one of the pregnancy outputs already mentioned in category B), 1: chronically ill mothers, 2: mothers without chronic diseases.

The frequency of complications varied between the groups as there were statistically significant differences in the frequency of infections (χ^2^ = 4.216; df =1; p < 0.05) and vaginal hemorrhages (χ^2^ = 5.537; df = 1; p < 0.05) but varied less so for acute diseases (χ^2^ = 3.831; df = 1; p = 0.05). Complications were more frequent in the healthy group than in the chronic disease group (Figure 4). The fewest complications occurred in Category C (multigravida, multiparous women, with an equal number of pregnancies and live births), and the highest frequency of complications occurred in primiparous women in Category B. When comparing primiparous and multiparous women, the latter group had more frequent complications and a statistically significant difference in acute diseases (χ^2^ = 6.698; df =1; p = 0.01).

In total, n_1_ = 297 and n_2_ = 947 women were hospitalized for less than 15 days and n_1_ = 65 and n_2_ = 137 women were hospitalized for 15 days or more (|t| = 2.795; p < 0.01). Pregnant women in the chronic disease group were hospitalized an average of two days longer (x = 11d; σ = 14.47) than women in the healthy group (x = 8.9 d; σ = 12.21).

**Figure 5:**
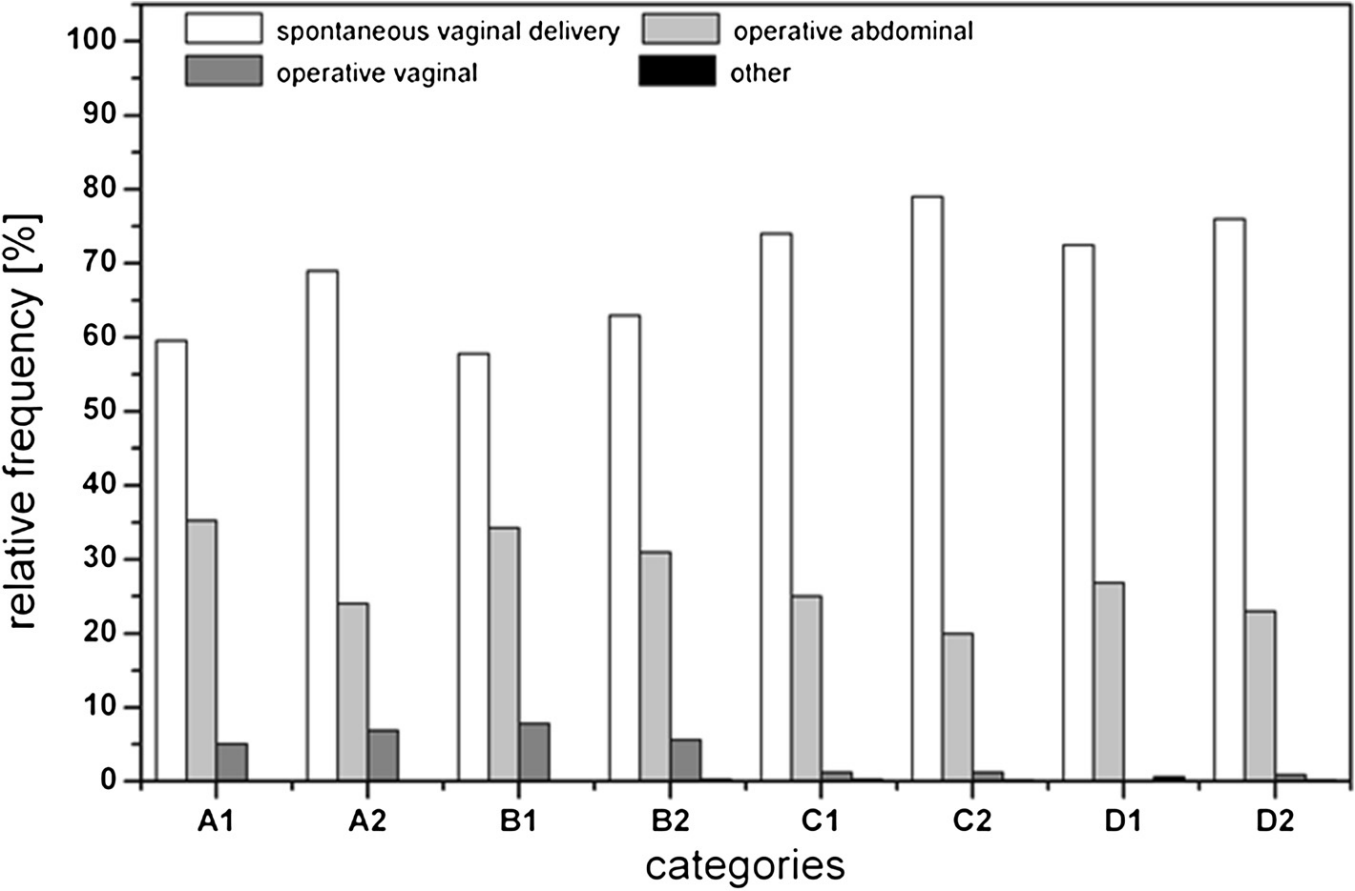
**Birth mode of women who participated in SNiP plot against chronic diseases as well as pariety and gravidity.** A: primigravidae, primiparae, B: multigravidae, primiparae (women who had have at least one abruption, extrauterine pregnancy, abortion or stillbirth and who participated in SNiP with their first child), C: multigravidae, multiparae (the number of pregnancies correlates with the number of life births), D: multigravidae, multiparae (women who had have at least one of the pregnancy outputs already mentioned in category B), 1: chronically ill mothers, 2: mothers without chronic diseases.

The spontaneous vaginal delivery was in the preferred birth modus in both groups with highest rates among healthy multiparous women (Figure 5); however, there was a significant difference between the groups (χ^2^ = 26.370; df =3; p < 0.01) as women with chronic disease delivered by Cesarean section more frequently than women in the healthy group. Primiparous women delivered more frequently via operative vaginal delivery than multiparous women.

### Perinatal parameters

**Figure 6:**
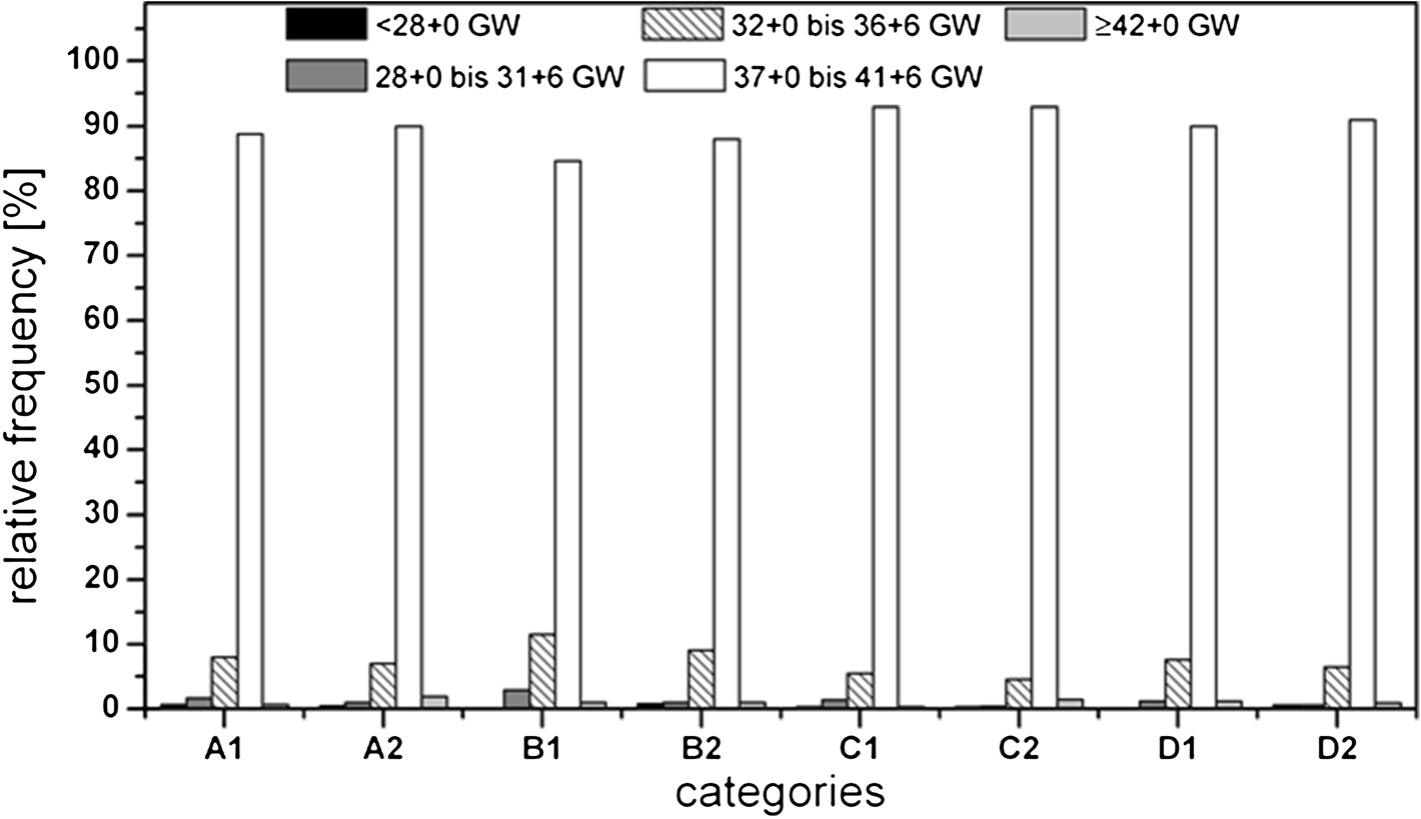
**Gestational age (GW: gestational week) plot against chronic diseases as well as pariety and gravidity. **A: primigravidae, primiparae, B: multigravidae, primiparae (women who had have at least one abruption, extrauterine pregnancy, abortion or stillbirth and who participated in SNiP with their first child), C: multigravidae, multiparae (the number of pregnancies correlates with the number of life births), D: multigravidae, multiparae (women who had have at least one of the pregnancy outputs already mentioned in category B), 1: chronically ill mothers, 2: mothers without chronic diseases.

The youngest gestational age in the chronically ill group was 24 + 1 and the oldest was 42 + 1. In the healthy group, the gestational age ranged from 22 + 1 to 42 + 5 gestational weeks (Figure 6). A statistically significant difference in gestational age was only observed between categories A and C in the chronic disease group (|t| = 1.181; p < 0.05). In the chronic disease group, there were significant differences between categories A and B (|t| = 2.089; p < 0.05), B and C (|t| = 3,129; p < 0.05) and C and D (|t| = 2.961; p < 0.05). There was a statistically significant difference in favor of healthy mothers when the gestational ages were compared between the two groups (|t| = 3.380; p < 0.05). A total of 10.2% of all infants born to participants with chronic diseases and 8.1% of all infants born to healthy mothers were born prior to 37 gestational weeks. This means that every tenth woman with at least one chronic disease gave birth to a premature infant, while only one in every 13 woman in the healthy control group gave birth to a premature infant (χ^2^ = 5.091; df = 1; p < 0.05). Women in categories B and D (e.g., the number of pregnancies is not equal to the number of live births) delivered an average of one to two days earlier than women in categories A and C (i.e., the number of pregnancies equals the number of live births). In the group of women with chronic diseases, 12 of the twin births (60%) were premature. In the healthy group, 28 of the twin births (55%) were premature; however, a chi-square test did not reveal any statistically significant differences (χ^2^ = 0.304, df =1; p > 0.05).

On average, the newborns of primiparous women had a lower birth weight, birth length and head circumference than those of multiparous women. The birth weight range was between 336 and 5050 grams in the group of infants born to mothers with chronic diseases and between 450 and 5490 grams in the control group (not statistically significant). Comparable ranges and significant differences were reported for birth length (26 until 59 cm; |t| = 2.744; p < 0.01) and head circumference (19 until 40 cm; |t| = 2.316; p < 0.05) between the two groups. We also observed a significant difference in the 5 minute Apgar scores for primiparous and multiparous women (|t| = 2.692; p < 0.05) but not for the comparison between chronically ill and healthy women (|t| = 1.447; p > 0.05). In categories A, C and D, less than 1% of the infants had a poor prognostic 5 minute Apgar score, compared to 1.9% of the infants of mothers with chronic diseases in category B (primiparous, multigravida).

**Figure 7:**
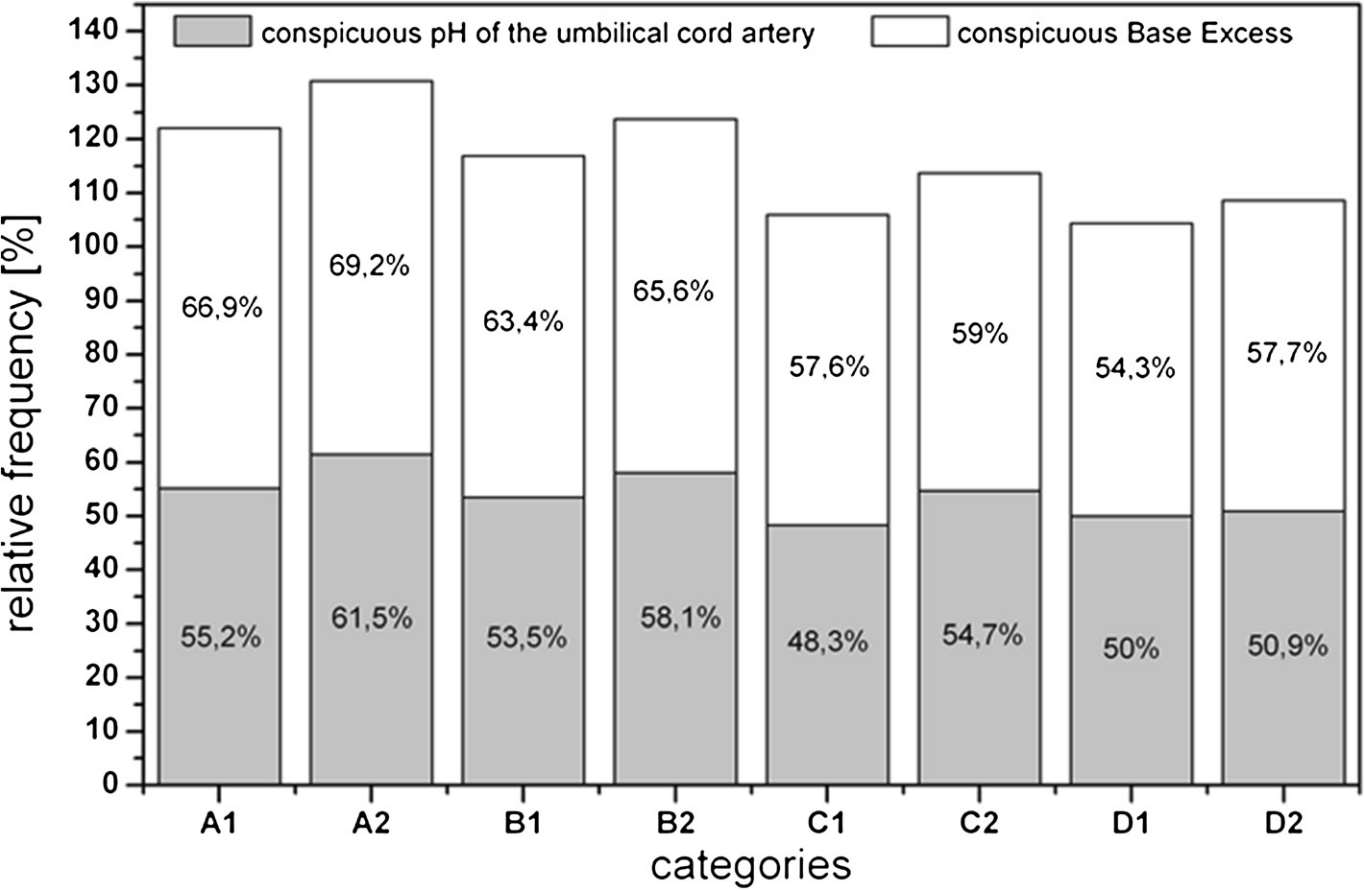
**Pathologic pH of the umbilical cord artery and of Base Excess plot against chronic diseases as well as pariety and gravidity. **A: primigravidae, primiparae, B: multigravidae, primiparae (women who had have at least one abruption, extrauterine pregnancy, abortion or stillbirth and who participated in SNiP with their first child), C: multigravidae, multiparae (the number of pregnancies correlates with the number of life births), D: multigravidae, multiparae (women who had have at least one of the pregnancy outputs already mentioned in category B), 1: chronically ill mothers, 2: mothers without chronic diseases.

Abnormalities in the pH-values of the umbilical cord artery and of the base excess are shown in Figure 7 according to maternal categories. The data shows that infants born to healthy mothers are better able to adapt than those born to ill mothers. The chi-square test is significant for the pH-value but not for the base excess (pH-value: χ^2^ = 10.095; df =1; p < 0.01; Base Excess: χ^2^ = 2.085; df = 2; p > 0.05). Multiparous women with chronic diseases had a lower proportion of infants with abnormal pH-values and/or base excess, whereas healthy primiparous women had higher rates.

**Figure 8:**
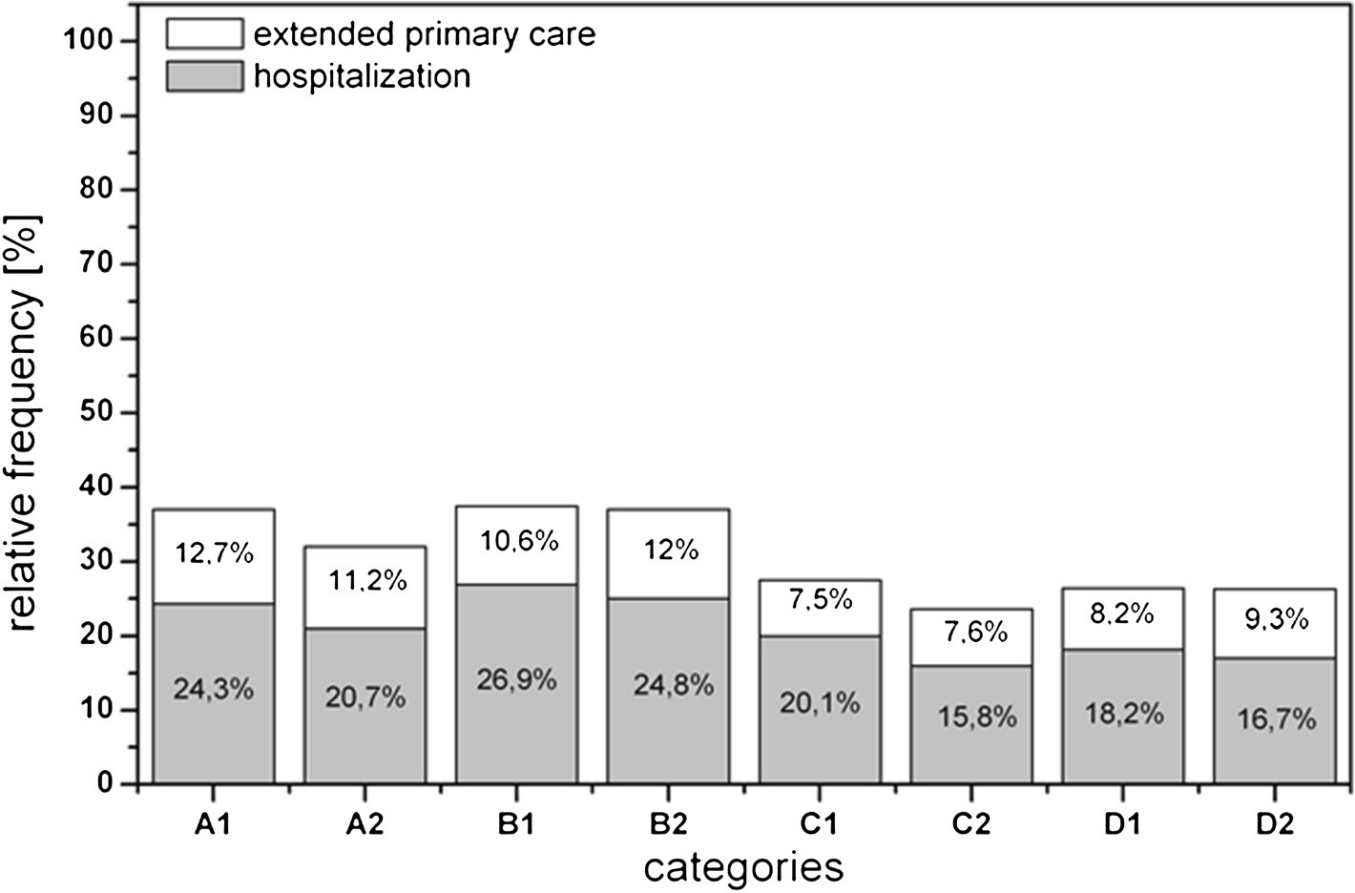
**Frequency of extended primary care and of hospitalisation plot against chronic diseases as well as parity and gravidity.** A: primigravidae, primiparae. B: multigravidae, primiparae (women who had have at least one abruption, extrauterine pregnancy, abortion or stillbirth and who participated in SNiP with their first child), C: multigravidae, multiparae (the number of pregnancies correlates with the number of life births), D: multigravidae, multiparae (women who had have at least one of the pregnancy outcomes already mentioned in category B), 1: chronically ill mothers, 2: mothers without chronic diseases.

Figure 8 provides the percentage of infants who received either extended primary care and/or who were hospitalized after birth. Significant differences are observed for hospitalization (χ^2^ = 7.294; df =1; p < 0.01) but not for extended primary care (χ^2^ = 0.114; df = 2; p > 0.05). On average, the hospitalization rate was higher among infants born to ill mothers (22.4%) than healthy ones (19.5%).

## Discussion

The aim of this study was to analyze the prevalence of chronic diseases in women of childbearing age and investigate the influence of these diseases on pregnancy and birth. This analysis is the first population-based study in which all chronic diseases could be taken into consideration. In the SNiP, every fifth pregnant woman suffers from at least one chronic disease, and higher prevalence rates have been reported in the literature. In an American study analyzing 6.294 women of childbearing age, 26.6% of the participants had one of the more prevalent chronic diseases. In contrast, 39.1% of all women who were not pregnant reported that they were chronically ill [8]. The population-based prevalences rates in the SNiP data are consistently lower than those found in the literature. However, in this study the prevalence rates in young mothers have been contrasted to all women in childbearing age. The prevalence of some chronic diseases is lower during pregnancy (e.g., epilepsy, asthma, and some other immune mediated diseases); however, some participants may not have provided full information on chronic diseases prior to their pregnancy. A bias resulting from a selective referral of chronically ill pregnant women to specialized centers outside the study region is most likely small because the study region is the medical center of a larger region and contains the regional secondary and tertiary care facilities. The different prevalence rates reported in the literature can be partly explained by differences in study design, lack of standardized diagnostics and regional differences (ethnicity, rurality, climatic influences, etc.).

The assumption that women with chronic diseases in childbearing age have a reduced fertility could not be proven by the SNiP data. Fertility may not be affected by some chronic diseases (like bronchial asthma [9]), while others, such as epilepsy, are associated with limited fertility [10,11]. Possible future research could include studies that are not population-based but rather focus on specific diseases at specialized centers.

Finally, the definition of the term “fertility” is important. In this analysis, the maternal age at the point of birth as well as quantity and quality of prior pregnancies were used in the operationalization of “fertility”. The results could have been different if the definition were based on the number of cycles prior to conception or laboratory results. Furthermore, other factors known to reduce fertility have to be included, e.g., nicotine consumption [12]. In our analysis, chronic disease has a significant influence on pregnancy and pregnancy outcomes; however, it is less significant than often supposed. We could not show significant socio-demographic differences between the two groups with respect to family status, ethnic background and pregnancy planning. Indeed, socio-demographics explain differences in education, qualification, occupation, and income. In our analysis, these differences were statistically significant, but the absolute values were small. Because the women in the chronic disease group were on average one year older at the time of childbirth, it may be possible that they already had more income and higher qualifications. The difference in age is also described in the literature [8]. The course of pregnancy was on average more complicated in the group of healthy women than for women with chronic diseases with respect to vaginal hemorrhages, acute diseases and infections.

The total time of hospitalization during pregnancy was longer for women with chronic diseases than for healthy women. In conclusion, we cannot state with certainty whether the pregnancy course is more complicated for chronically ill women. As hypothesized, women with chronic diseases had a higher frequency of caesarean sections; however, the physiologically spontaneous birth was still the most frequent birth mode in both groups. As previously mentioned, a comparison of specific diseases would allow for more detailed estimations. A total of 10.2% of newborns born to mothers with chronic diseases were premature (versus 8.1% of newborns of healthy mothers). The higher rate of premature births for women may explain some of the difference in the frequency of caesarean sections.

While there were no significant differences in the 5 minute Apgar scores, the base excess extended newborn primary care, and the pH of the umbilical cord artery were significantly more often pathological in the healthy population. The higher frequency of caesarean sections in women with chronic diseases could be a possible explanation for this somewhat unexpected observation. Infants that were born by caesarean section had less stress under birth and therefore better laboratory values. Newborns of mothers with chronic diseases were more often hospitalized. In future studies, birth outcomes should be investigated more comprehensively, including the physiological and psychological development of the infant.

Furthermore, we have observed that a higher percentage of twin births occurred for chronically ill mothers. Based on the analysis by Hellin (established in 1895) that the frequency of twin births = 1:85 pregnancies (1.2%), there should have been 14 twin pregnancies in the chronic disease group (observed 20) and 50 in the healthy group (observed 51). One explanation could be that the women in the chronic disease group were slightly older, which is associated with a higher tendency for multiple births. Another possible reason could be the higher frequency of preconception hormone therapies or artificial inseminations in the chronic disease group due to reduced fertility. However a more detailed analysis with a larger sample is required to determine if there is a link between chronic illness and multiple births.

### Limitations of the analysis

There are limitations to this analysis that could decrease validity. First, the limitation of the study design is, that data about the pregnancy itself was assessed in a retrospective manner. It was taken from the women’s physician’s records. This might be a threat to the validity of the data since (a) individual differences between physician’s can be expected but not identified and controlled for and. However, the physician’s records seem to be a more valid information system than asking women retrospectively at time of birth about pregnancy related diagnoses, examinations and other obstetric factors. It is not clear whether these differences will yield different results.

Secondly, not all pregnancies in women with chronic diseases are high-risk pregnancies. However, the aim of the study is to take into account every chronic condition, and to give an accurate overview of the prevalence of chronic diseases on a population basis. To analyse the relation between chronic disease and pregnancy complications more detailed analyses are necessary and should be focused on categories of disease secondly.

## Conclusions

This analysis is the first population-based study in which the prevalences of all major chronic diseases were included and not only the most common ones. Every fifth women in the study region suffered from at least one chronic disease. In addition, the perinatal outcome seems to be less favorable for infants of women with chronic diseases.

## Competing interests

The authors declare that they have no competing interests.

## Authors’ contributions

IK was responsible for data collection and writing of the manuscript, AEL was responsible for data collection, quality assessment and classification and for writing and revising the manuscript, JPH, CF, WH and JRT originally conceived of the study and contributed to scientific interpretation of the results and manuscript revision, HL contributed to scientific interpretations and manuscript revision. All authors read and approved the final manuscript.

## Pre-publication history

The pre-publication history for this paper can be accessed here:

http://www.biomedcentral.com/1471-2393/14/75/prepub
